# Differences in the prevalence and risk factors of osteoporosis in chinese urban and rural regions: a cross-sectional study

**DOI:** 10.1186/s12891-023-06147-w

**Published:** 2023-01-19

**Authors:** Miao Zheng, Yanan Wan, Gongwen Liu, Yan Gao, Xiaoqun Pan, Wanxi You, Donglan Yuan, Jianxin Shen, Junhua Lu, Xia Wang, Gangfeng Zheng, Zhiqiang Han, Xinlin Li, Kai Chen, Xiaoxi Xing, Dong Zhang, Chengwei Weng, Qi Wei, Yongqing Zhang, Hua Lin

**Affiliations:** 1 The Osteoporosis Clinical Center, The Second Affiliated Hospital of Soochow University, Suzhou, 215004 China; 2 Department of Non-Communicable Chronic Disease Control, Jiangsu Provincial Center for Disease Control and Prevention, Nanjing, 210009 China; 3 Department of Orthopedics, Suzhou TCM Hospital Affiliated to Nanjing University of Chinese Medicine, Suzhou, 215004 China; 4 Department of Orthopedics, The First People’s Hospital of Kunshan, Kunshan, 215300 China; 5 Department of Chronic Disease Prevention and Control, Luhe District Center for Disease Control and Prevention, Nanjing, 215200 China; 6 Department of Nuclear Medicine, The First Affiliated Hospital of Nanjing Medical University, Nanjing, 210029 China; 7 Department of Chronic Disease Prevention and Control, Suzhou Wujiang District Center for Disease Control and Prevention, Suzhou, 215200 China; 8 Department of Chronic Disease Prevention and Control, Chongchuan District Center for Disease Control and Prevention, Nantong, 226001 China; 9 Department of Osteoporosis, Third Affiliated Hospital of Nantong University, Nantong, 226000 China; 10 Department of Chronic Diseases, Jingjiang Center for Disease Control and Prevention, Jingjiang 214500, Beijing, China; 11 Taizhou Hospital of Traditional Chinese Medicine, Taizhou, 225300 China; 12 Department of Chronic Diseases, Nantong Center for Disease Control and Prevention, Nantong, 214500 China; 13 XuYi People’s Hospital, Huaian, 211700 China; 14 Quanshan District Center for Disease Control and Prevention, Xuzhou, 221002 China; 15 Department of Orthopedics, Affiliated Drum Tower Hospital, Medical School of Nanjing University, Nanjing, 210008 China

**Keywords:** Osteoporosis, Prevalence, Urban and rural, Influencing factor

## Abstract

**Background:**

Bone mineral density (BMD) and prevalence of osteoporosis may differ between urban and rural populations. This study aimed to investigate the differences in BMD characteristics between urban and rural populations in Jiangsu, China.

**Methods:**

A total of 2,711 participants aged 20 years and older were included in the cross-sectional study. Multistage and stratified cluster random sampling was used as the sampling strategy. BMD was measured by the method of dual-energy x-ray absorptiometry (DXA). Data were collected through questionnaires/interview. BMD values at the lumbar spine (L1-L4), femoral neck, total hip, and greater trochanter were collected. Descriptive statistics were used to demonstrate the characteristics of urban and rural participants. Multivariate logistic regression analysis was utilized to analyze the factors that may be associated with osteoporosis in urban and rural populations.

**Results:**

Of these participants, 1,540 (50.49%) were females and 1,363 (42.14%) were from urban. The prevalence of osteoporosis in urban and rural populations was 5.52% and 10.33%, respectively. In terms of gender, the prevalence of osteoporosis was 2.68% in males and 13.82% in females. For menopausal status, the prevalence of osteoporosis was 30.34% in postmenopausal females and 4.78% in premenopausal females. In urban populations, older age [adjusted odds ratio (AOR) = 2.36, 95%CI, 2.35–2.36), hypertension (AOR = 1.37, 95%CI, 1.36–1.37), unmarried (AOR = 4.04, 95%CI, 3.99–4.09), smoking everyday (AOR = 2.26, 95%CI, 2.23–2.28), family history of osteoporosis (AOR = 1.66, 95%CI, 1.65–1.67), dyslipidemia (AOR = 1.05, 95%CI, 1.04–1.05), and higher β-crosslaps (β-CTX) level (AOR = 1.02, 95%CI, 1.02–1.02) were associated with an increased risk of osteoporosis, while males (AOR = 0.04, 95%CI, 0.04–0.04), higher education level (AOR = 0.95, 95%CI, 0.95–0.95), and aquatic product intake (AOR = 0.99, 95%CI, 0.99–0.99) were related to decreased risk of osteoporosis. Similar results were also observed in rural populations, and (all *P* < 0.05).

**Conclusion:**

The prevalence of osteoporosis in rural populations was higher than that in urban populations, and the factors associated with the risk of osteoporosis were similar in urban and rural populations.

**Supplementary Information:**

The online version contains supplementary material available at 10.1186/s12891-023-06147-w.

## Background

Osteoporosis is a systemic skeletal disease characterized by increased bone fragility and fracture susceptibility due to low bone mass and degeneration of bone tissue microarchitecture [1]. Osteoporosis contributes a significant disease burden globally, with the number of deaths and disability-adjusted life-years due to low bone mineral density (BMD) increasing globally from 207,367 and 8,588,936 in 1990 to 437,884 and 16,647,466 in 2019 [2]. Differences in the incidence and prevalence of osteoporosis worldwide are difficult to determine due to underdiagnosis [3]. It has been estimated that 10.3% of the United States adults aged 50 years and older have osteoporosis [4]. The age‐standardized prevalence of osteoporosis in Chinese men and women aged 50 years and older was 6.46% and 29.13%, respectively [5]. A systematic review and meta-analysis showed that the global pooled prevalence of osteoporosis was 18.3% [6].

The risk of osteoporosis is related to many factors including advancing age, ethnicity, female gender, underweight, family history of osteoporosis, smoking, vitamin D deficiency, physical inactivity, and low estrogen status [7–10]. However, previous studies on the prevalence and risk factors of osteoporosis were mainly based on urban populations [5, 8, 9]. Some evidence suggested that the prevalence of osteoporosis may differ between urban and rural populations [11–14]. Tanaka et al. [12] and Sanders et al. [15] found that the prevalence of osteoporosis or fracture was significantly higher in the urban region than that in the rural region. On the contrary, some studies supported the results that rural populations had a significantly higher prevalence of osteoporosis than urban populations [14, 16]. Differences in osteoporosis between urban and rural populations may require more research to explore. Furthermore, the epidemiological characteristics of osteoporosis in the Chinese population were poorly understood compared to Western countries [17].

The present study aimed to investigate the differences in bone mineral density (BMD) and prevalence of osteoporosis between urban and rural populations in Jiangsu, China. Factors that may be associated with osteoporosis in urban and rural populations were explored.

## Methods

### Study population

This cross-sectional study population was collected from Jiangsu Province as part of a national osteoporosis epidemiological survey (2017) in China between January 2017 and April 2018. The national osteoporosis epidemiological survey (2017) was conducted in 11 provincial administrative units in China. Each administrative unit selected 4 regions, a total of 44 regions were investigated. Jiangsu Province is located in the eastern of China, with a predominantly plain terrain, and is an economically developed region of China. Four cities in Jiangsu Province were selected to represent urban (Nanjing-Liuhe District and Nantong-Gangzha District) and rural (Suzhou-Wujiang District and Taizhou-Jingjiang) areas respectively. Eligible participants were aged ≥ 20 years and had complete BMD measurement data. The exclusion criteria were as: (1) participants diagnosed with metabolic bone disease such as hyperthyroidism, hyperparathyroidism, renal failure, malabsorption syndrome, alcoholism, chronic colitis, multi-myeloma, leukemia, and chronic arthritis; (2) pregnant participants.

### Sample size and sampling

The sample size calculation of the national survey (2017) was used for this study. The sample population was divided into 20–39 years and 40 years and above based on age, with the 20–39 years group being used to investigate peak bone mass in the Chinese population and the 40 years and above group being used to assess the prevalence of osteoporosis.

The sample size for people aged 40 years and above was calculated using the prevalence of osteoporosis:

$$N=deff\frac{{u}_{\alpha }^{2}p(1-p)}{{d}^{2}}$$

According to previous research [17], the estimated *p* value of the prevalence of osteoporosis in this study is 0.132. The value of α is 0.05 (two-sided), the value of u_α_ is 1.96, the value of d is 0.0198 (relative error = 0.15, d = 0.15*0.132), and the design effect is 3. In addition, the stratification factors gender (male and female) and region (urban and rural) were considered in the sample size calculation. According to the formula, the average sample size of each layer (4 layers) is 3,369 people. Taking into account the above stratification factors and the 80% response rate, the minimum total sample size was calculated to be 16,845 people for the 40 years and above group, and each provincial administrative unit (11 units) sampled 1,532 people.

The sample size for people aged 20–39 years was calculated using peak BMD:

$${N=\left(\frac{{u}_{\alpha }\sigma }{\delta }\right)}^{2}$$

The value of α is 0.05 (two-side), and the value of u_α_ is 1.96. σ is the overall standard deviation, and according to previous studies [18, 19], the standard deviation of BMD in people aged 20–39 years ranged from 0.090 g/cm^2^ to 0.196 g/cm^2^, and σ was taken as 0.196 for this study. δ is the allowable error and was taken as 25% of the standard deviation (taken as 0.090). Taking into account the gender, region, and age (20–29, 30–39 years) factors and the 80% response rate, the minimum total sample size was calculated to be 2,790 people for the 20–39 years group, and each provincial administrative unit was sampled 254 people. Therefore, the minimum total sample size required for each provincial administrative unit was 1,786, and a total of 2,710 participants were included in this study to meet the adequate sample size. In addition, our sample size was sufficient for statistical analysis according to the events per variable (EPV) rule [20].

The sampling method of this study was multistage and stratified cluster random sampling. In each survey area (4 areas), 4 towns/streets were randomly selected by cluster sampling method proportional to population size (PPS), and 2 administrative villages/neighborhood committees were randomly selected from each town/street (PPS sampling). One resident group for each administrative village/neighborhood committee was selected at random (each resident group should include at least 50 participants aged 40 years and above and 8 participants aged 20–39 years).

### Data collection

The primary outcome of this study was prevalence of osteoporosis. All participants received a face-to-face interview and physical examination, which was conducted by investigators trained in standard research protocols. A standardized questionnaire was used to assess risk factors for osteoporosis, including sociodemographic factors, lifestyle factors, dietary intake, physical activity, and family history of osteoporosis or fragility fracture. Information of participants were collected including gender (male and female), age (20–29, 30–39, 40–49, 50–59, 60–69, 70–79, and 80–89 years), area (urban and rural), body mass index (BMI), systolic blood pressure (SBP), diastolic blood pressure (DBP), heart rate, BMD levels [lumbar spine (L1, L2, L3, L4), the greater trochanter, and total hip], ethnicity (Han and others), education levels (< high school, high school, and college and above), marital status (unmarried, married, cohabitation, widowed, and divorced), income, expenditure, smoking (everyday, not every day, smoking before but not present, and never), drinking (never, sometimes, often but not exceeding the norm, often and beyond the norm), family history of osteoporosis (yes, no, and unknown), diet (rice/pasta, tuber, pork, aquatic product, vegetables, and eggs), physical activity (high-intensity and moderate-intensity), activity duration, sleep duration, fasting plasma glucose, triglyceride, total cholesterol, low density lipoprotein cholesterol (LDL-C), high density lipoprotein cholesterol (HDL-C), calcium, phosphorus, 25-hydroxyvitamin D (25(OH)D), β-crosslaps (β-CTX), and procollagen type I N-terminal propeptide (PINP). BMI was divided into three types, including underweight (BMI < 18.5 kg/m^2^), normal weight (18.5 kg/m^2^ ≤ BMI < 24.0 kg/m^2^), and overweight (BMI ≥ 24.0 kg/m^2^) [21]. Hypertension was defined as SBP ≥ 140 mm Hg, and/or DBP ≥ 90 mm Hg, and/or use of antihypertensive medications within the past two weeks [22]. Hyperglycemia was defined as fasting plasma glucose ≥ 6.11 mmol/L [23]. Dyslipidemia was defined based on current lipids levels or the use of anti-dyslipidemia medications within the past two weeks. The cut-off values were 6.22 mmol/L for higher total cholesterol, 4.14 mmol/L for higher LDL-C, 1.04 mmol/L for lower HDL-C, and 2.26 mmol/L for higher triglyceride [24].

### BMD measurements and the definition of osteoporosis

BMD was measured by the method of dual energy x-ray absorptiometry (DXA) using GE Lunar DXA scanners (Prodigy or iDXA; GE Healthcare, Waukesha, WI, USA). The measurement of BMD was performed first by scanning the lumbar spine, and then by scanning the left proximal femur including the femoral neck, total hip, and greater trochanter. The quality control process was carried out based on the manufacturer’s operating manual. In addition, the unified European spine phantom (ESP) was scanned 10 times to calibrate each DXA scanner and repositioned for each scan.

Osteoporosis and low BMD were defined according to World Health Organization criteria [25]. Osteoporosis was defined as a T-score ≤ -2.5 standard deviation (SD), and low BMD was defined as a -1 SD < T-score < -2.5 SD. T-scores were calculated as (measured BMD—peak BMD)/SD. The peak BMD was defined as the maximal sex-specific mean BMD. In addition, T-scores were calculated based on peak bone mass determined for males and females, respectively.

### Laboratory testing

Blood biochemistry and bone turnover indicators of participants including fasting plasma glucose, triglyceride, total cholesterol, LDL-C, HDL-C, calcium, phosphorus, 25(OH)D, β-CTX, and PINP were measured by the third-party laboratory according to the relevant technical manual. Fasting venous blood of participants was collected using 5 ml vacuum coagulant tube and 2 ml Na-F anticoagulant tube, respectively. Blood samples from 2 ml Na-F anticoagulant tube were directly centrifuged and 0.6–1.0 ml of plasma was collected and dispensed into 1.5 ml blood glucose testing tube and frozen at -20℃ for fasting blood glucose testing. Blood samples in 5 ml vacuum coagulant tube were used to test other indexes, centrifuged after 45 min at room temperature, and the serum was collected and divided into two tubes, one for testing (at least 1.5 ml serum) and the other for storage, and both frozen at -20℃.

### Statistical analysis

Continuous variables were described as mean and standard error (S.E.), and weighted independent samples t-test was used for comparison between groups. Categorical variables were expressed as numbers and percentages (n (%)), and the comparison between groups used weighted Chi-square test. All percentages were weighted results due to the sampling method of multistage and stratified cluster random sampling. Multivariate logistic regression analysis was utilized to analyze the factors that may be associated with osteoporosis in urban and rural populations. Variables with statistically significant differences (*P* < 0.05) on binary analysis were included in multivariate logistic regression analysis using stepwise regression method (backward). Variables with *P* ≥ 0.05 in stepwise regression were excluded step by step in each fitting process. Statistical analysis was performed by SAS 9.4 software (SAS Institute Inc., Cary, NC, USA), and bar chart were drawn using GraphPad Prism 8 software (GraphPad Software, San Diego, California, USA). *P* < 0.05 was considered statistically significant. Adjusted odds ratio (AOR) with 95% confidence interval (CI) were used for association assessment.

## Results

### Characteristics of participants

Information on 2,780 participants was collected, and after excluding 69 participants with missing bone mass measurement data, 2,711 participants were included in this study. Table 1 shows the characteristics of all participants. Of these 2,711 participants, 1,540 (50.49%) were females, 1,363 (42.14%) were from urban, and 2,346 (59.69%) were aged ≥ 40. Of the 1,540 females, 946 (35.34%) were postmenopausal. The number of patients with osteoporosis and low BMD was 371 (8.30%) and 1,053 (33.21%), respectively. The mean (S.E.) BMD values of participants were 0.91 (0.01) g/cm^2^ for the lumbar spine L1, 0.99 (0.01) g/cm^2^ for the L2, 1.05 (0.01) g/cm^2^ for the L3, 1.05 (0.01) g/cm^2^ for the L4, 0.64 (0.00) g/cm^2^ for the greater trochanter, and 0.88 (0.01) g/cm^2^ for the total hip.

**Table 1:** Characteristics of study populations

Variables	Total (*n* = 2,711)	Urban (*n* = 1,363)	Rural (*n* = 1,348)	Statistics	*P*
Gender, n (%)				χ^2^ = 0.211	0.646
Males	1171 (49.51)	590 (50.68)	581 (48.67)		
Females	1540 (50.49)	773 (49.32)	767 (51.33)		
Age (years), n (%)				χ^2^ = 7.976	0.240
20–29	181 (21.32)	91 (25.18)	90 (18.50)		
30–39	184 (18.99)	93 (20.29)	91 (18.05)		
40–49	578 (23.28)	302 (22.05)	276 (24.18)		
50–59	686 (16.60)	347 (15.36)	339 (17.50)		
60–69	798 (11.06)	407 (9.75)	391 (12.02)		
70–79	255 (7.75)	113 (6.43)	142 (8.71)		
80–89	29 (1.00)	10 (0.94)	19 (1.04)		
BMI (kg/m^2^), mean (S.E)	24.36 (0.15)	24.12 (0.27)	24.54 (0.16)	t = -1.35	0.177
BMI (kg/m^2^), n (%)				χ^2^ = 7.389	0.025
Underweight (< 18.5)	58 (3.21)	22 (2.37)	36 (3.82)		
Normal (18.5–23.9)	1078 (46.06)	532 (52.02)	546 (41.72)		
Overweight (≥ 24.0)	1575 (50.73)	809 (45.61)	766 (54.47)		
Osteoporosis, n (%)				χ^2^ = 16.188	< 0.001
No	2340 (91.70)	1202 (94.48)	1138 (89.67)		
Yes	371 (8.30)	161 (5.52)	210 (10.33)		
Low BMD, n (%)				χ^2^ = 5.232	0.022
No	1658 (66.79)	861 (71.91)	797 (63.05)		
Yes	1053 (33.21)	502 (28.09)	551 (36.95)		
Lumbar spine L1 (g/cm^2^), mean (S.E)	0.91 (0.01)	0.95 (0.01)	0.87 (0.01)	t = 7.20	< 0.001
Lumbar spine L2 (g/cm^2^), mean (S.E)	0.99 (0.01)	1.02 (0.01)	0.96 (0.01)	t = 4.98	< 0.001
Lumbar spine L3 (g/cm^2^), mean (S.E)	1.05 (0.01)	1.09 (0.01)	1.02 (0.01)	t = 5.77	< 0.001
Lumbar spine L4 (g/cm^2^), mean (S.E)	1.05 (0.01)	1.08 (0.01)	1.03 (0.01)	t = 3.66	< 0.001
Greater trochanter (g/cm^2^), mean (S.E)	0.64 (0.00)	0.65 (0.01)	0.63 (0.00)	t = 2.20	0.028
Total hip (g/cm^2^), mean (S.E)	0.88 (0.01)	0.88 (0.01)	0.87 (0.01)	t = 0.77	0.441

*BMI* body mass index, *BMD* bone mineral density

#### Prevalence of osteoporosis in different populations

**Fig. 1:**
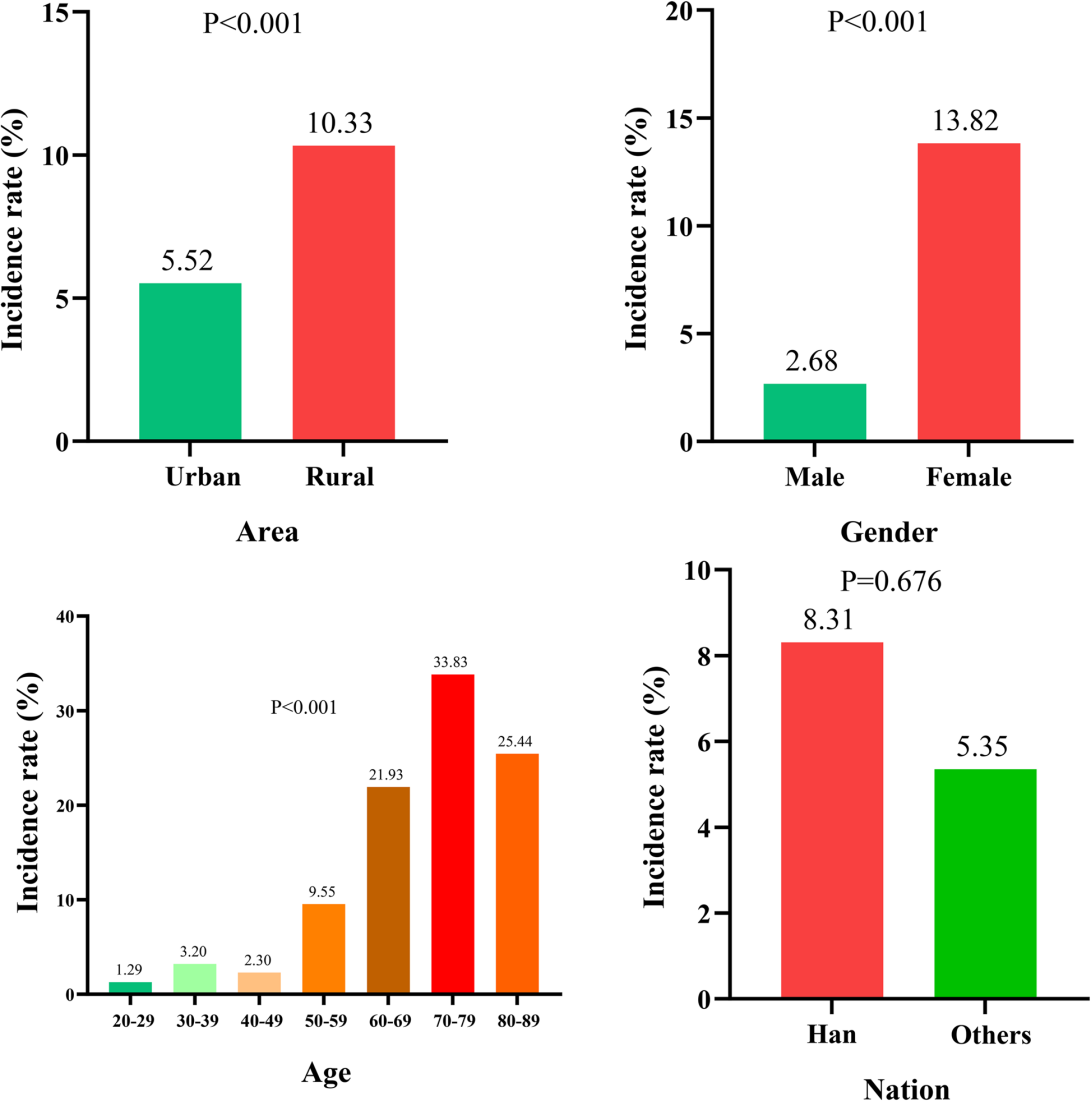
The prevalence of osteoporosis in different populations

**Fig. 2:**
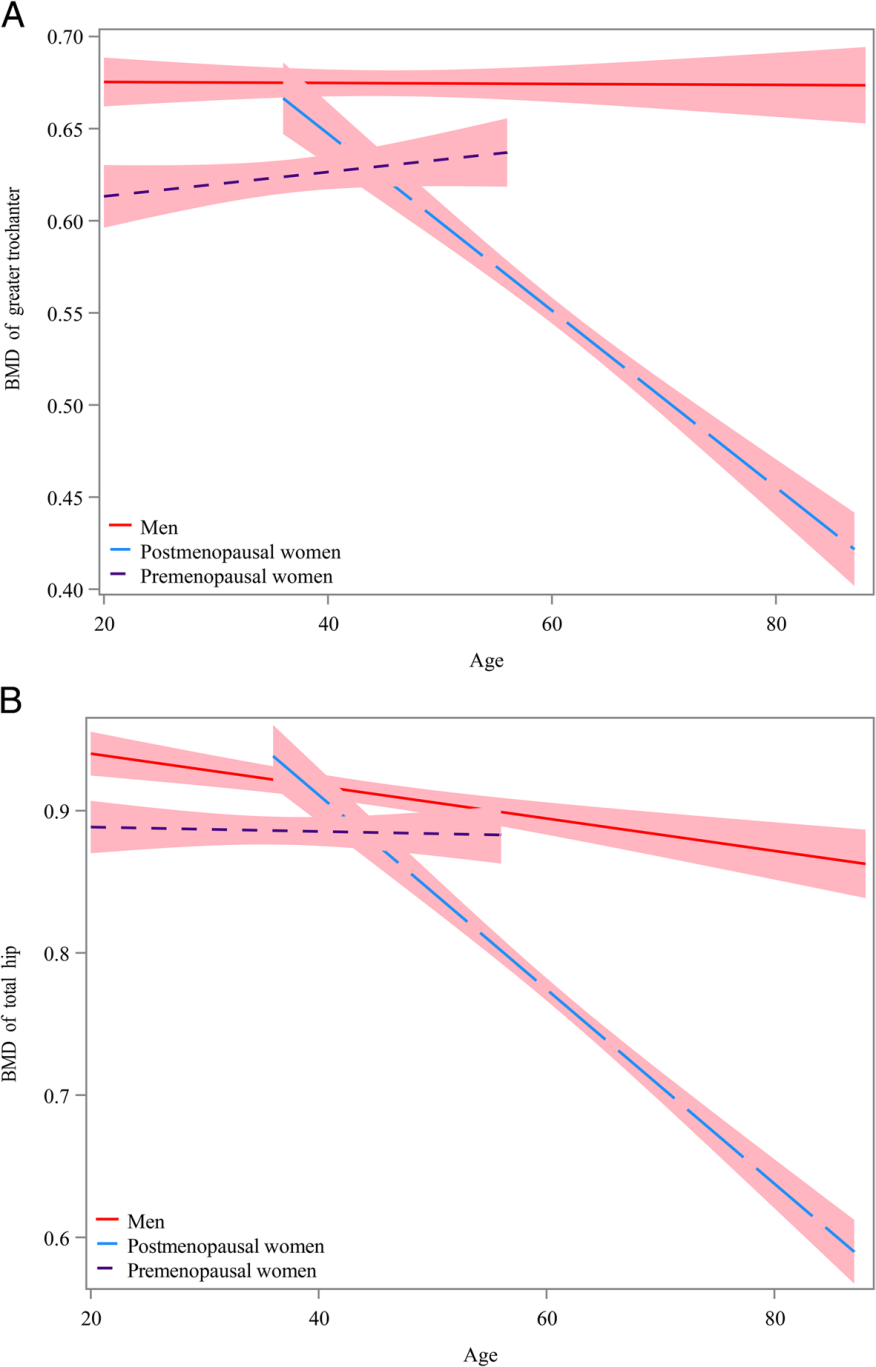
Curves of bone mineral density (BMD) with age at the greater trochanter and total hip in men, premenopausal women and postmenopausal women. **A** BMD of greater trochanter; **B** BMD of total hip

Figure 1 presents the prevalence of osteoporosis in different populations. The prevalence of osteoporosis in urban and rural populations was 5.52% (161 cases) and 10.33% (210 cases), respectively (*P* < 0.001). In different age groups, the prevalence of osteoporosis was 1.29% (3 cases) in the 20–29 years group, 3.20% (4 cases) in the 30–39 years group, 2.30% (14 cases) in the 40–49 years group, 9.55% (80 cases) in the 50–59 years group, 21.93% (184 cases) in the 60–69 years group, 33.83% (78 cases) in the 70–79 years group, and 25.44% (8 cases) in the 80–89 age group (*P* < 0.001). In terms of gender, the prevalence of osteoporosis was 2.68% (41 cases) in males and 13.82% (330 cases) in females (*P* < 0.001). In addition, the prevalence of osteoporosis was 30.34% (284 cases) in postmenopausal females and 4.78% (46 cases) in premenopausal females (*P* < 0.001). The curves of BMD with age at the greater trochanter and total hip in men, premenopausal women, and postmenopausal women were shown in Fig. 2. BMD of the greater trochanter showed a steady trend with age in men, a slow increase with age in premenopausal women, and a rapid decrease with age in postmenopausal women (Fig. 2A). BMD of the total hip showed a slow decline with age in both men and premenopausal women, while in postmenopausal women it showed a rapid decline with age. BMD of the total hip showed a slow decline with age in both men and premenopausal women, while in postmenopausal women it showed a rapid decline with age (Fig. 2B).

#### BMD characteristics of urban and rural female participants

According to menopause status, the BMD characteristics of urban and rural female participants were further analyzed (Table 2). The prevalence of osteoporosis in urban and rural postmenopausal females was 26.83% and 32.53%, respectively. Among urban females, the mean (S.E.) BMD values of postmenopausal females at the lumbar spine L1 [0.81 (0.01) vs. 1.00 (0.01) g/cm^2^], L2 [0.86 (0.01) vs. 1.07 (0.01) g/cm^2^], L3 [0.94 (0.01) vs. 1.15 (0.01) g/cm^2^], L4 [0.95 (0.01) vs. 1.12 (0.01) g/cm^2^], greater trochanter [0.57 (0.01) vs. 0.63 (0.01) g/cm^2^], and total hip [0.79 (0.01) vs. 0.89 (0.01) g/cm^2^] were significantly lower than that in premenopausal females (all *P* < 0.001). Similar results were found in rural postmenopausal females.

**Table 2:** BMD characteristics of urban and rural female participants

Variables	Urban (*n* = 773)		*P*	Rural (*n* = 767)		*P*
	**Postmenopausal (*n* = 476)**	**Premenopausal (*n* = 297)**		**Postmenopausal (*n* = 470)**	**Premenopausal (*n* = 297)**	
BMI (kg/m^2^), mean (S.E)	25.38 (0.25)	23.13 (0.32)	< 0.001	25.05 (0.23)	23.22 (0.30)	< 0.001
BMI (kg/m^2^), n (%)			< 0.001			< 0.001
Underweight (< 18.5)	6 (1.04)	6 (4.54)		8 (2.15)	16 (8.37)	
Normal (18.5–23.9)	161 (34.77)	163 (61.85)		173 (36.92)	145 (54.24)	
Overweight (≥ 24.0)	309 (64.19)	128 (33.61)		289 (60.93)	137 (37.39)	
Osteoporosis, n (%)			< 0.001			< 0.001
No	340 (73.17)	285 (97.89)		322 (67.47)	263 (93.23)	
Yes	136 (26.83)	12 (2.11)		148 (32.53)	34 (6.77)	
Low BMD, n (%)			< 0.001			< 0.001
No	245 (52.98)	226 (79.92)		229 (47.17)	201 (70.39)	
Yes	231 (47.02)	71 (20.08)		241 (52.83)	96 (29.61)	
Lumbar spine L1 (g/cm^2^), mean (S.E)	0.81 (0.01)	1.00 (0.01)	< 0.001	0.74 (0.01)	0.92 (0.01)	< 0.001
Lumbar spine L2 (g/cm^2^), mean (S.E)	0.86 (0.01)	1.07 (0.01)	< .001	0.81 (0.01)	1.02 (0.01)	< 0.001
Lumbar spine L3 (g/cm^2^), mean (S.E)	0.94 (0.01)	1.15 (0.01)	< 0.001	0.87 (0.01)	1.09 (0.01)	< 0.001
Lumbar spine L4 (g/cm^2^), mean (S.E)	0.95 (0.01)	1.12 (0.01)	< 0.001	0.91 (0.01)	1.09 (0.01)	< 0.001
Greater trochanter (g/cm^2^), mean (S.E)	0.57 (0.01)	0.63 (0.01)	< 0.001	0.53 (0.01)	0.62 (0.01)	< 0.001
Total hip (g/cm^2^), mean (S.E)	0.79 (0.01)	0.89 (0.01)	< 0.001	0.75 (0.01)	0.88 (0.01)	< 0.001

*BMI* body mass index, *BMD* bone mineral density

#### Differences between urban and rural patients with and without osteoporosis

Characteristics of urban patients with and without osteoporosis were shown in Supplement Table 1. The results indicated that there were significant differences in gender, age, SBP, education level, marital status, income, outcome, smoking, drinking, BMD at L1, L2, L3, L4, greater trochanter, and total hip, family history of osteoporosis, diet (rice/pasta and aquatic product), fasting plasma glucose, total cholesterol, LDL-C, HDL-C, hyperglycemia, dyslipidemia, calcium, phosphorus, β-CTX, and PINP between urban patients with and without osteoporosis (all *P* < 0.05).

Similar, the characteristics of rural patients with and without osteoporosis were shown in Supplement Table 2. Significant differences were observed in gender, age, BMI, SBP, education level, marital status, income, outcome, smoking, drinking, low BMD status, BMD at L1, L2, L3, L4, greater trochanter, and total hip, family history of osteoporosis, diet (pork and aquatic product), total cholesterol, LDL-C, HDL-C, dyslipidemia, calcium, phosphorus, β-CTX, and PINP between rural patients with and without osteoporosis (all *P* < 0.05).

#### Factors associated with osteoporosis

The multivariate logistic regression analysis of osteoporosis-related factors in urban and rural populations was displayed in Table 3. In urban populations, older age (AOR = 2.36, 95%CI, 2.35–2.36), hypertension (AOR = 1.37, 95%CI, 1.36–1.37), married status [divorce (AOR = 2.19, 95%CI, 2.14–2.23), widowed (AOR = 1.40, 95%CI, 1.39–1.41), and unmarried (AOR = 4.04, 95%CI, 3.99–4.09)], smoking status [smoking but not everyday (AOR = 3.76, 95%CI, 3.71–3.82), smoking everyday (AOR = 2.26, 95%CI, 2.23–2.28), and smoking before but not present (AOR = 4.34, 95%CI, 4.29–4.39)], family history of osteoporosis [yes (AOR = 1.66, 95%CI, 1.65–1.67) and unknown (AOR = 1.37, 95%CI, 1.36–1.38)], dyslipidemia (AOR = 1.05, 95%CI, 1.04–1.05), and higher β-CTX (AOR = 1.02, 95%CI, 1.02–1.02) were associated with an increased risk of osteoporosis. Males (AOR = 0.04, 95%CI, 0.04–0.04), higher education level (AOR = 0.95, 95%CI, 0.95–0.95), and diet [rice/pasta (AOR = 0.99, 95%CI, 0.99–0.99) and aquatic product (AOR = 0.99, 95%CI, 0.99–0.99)] were related to decreased risk of osteoporosis in urban populations. In terms of rural populations, older age (AOR = 1.69, 95%CI, 1.69–1.69), hypertension (AOR = 1.05, 95%CI, 1.05–1.06), family history of osteoporosis [yes (AOR = 1.15, 95%CI, 1.15–1.16) and unknown (AOR = 1.49, 95%CI, 1.49–1.50)], dyslipidemia (AOR = 1.15, 95%CI, 1.15–1.15), and higher β-CTX (AOR = 1.02, 95%CI, 1.02–1.02) were linked to higher risk of osteoporosis, while males (AOR = 0.21, 95%CI, 0.21–0.21), higher education level (AOR = 0.94, 95%CI, 0.94–0.94), diet [pork (AOR = 0.99, 95%CI, 0.99–0.99) and aquatic product (AOR = 0.99, 95%CI, 0.99–0.99)] were associated with a decreased risk of osteoporosis.

**Table 3:** Multivariate logistic regression analysis of osteoporosis-related factors in urban and rural populations

Variables	Urban		Rural	
	**AOR (95%CI)**	***P***	**AOR (95%CI)**	***P***
Gender				
Male	0.04 (0.04–0.04)	< 0.001	0.21 (0.21–0.21)	< 0.001
Female	Ref		Ref	
Age	2.36 (2.35–2.36)	< 0.001	1.69 (1.69–1.69)	< 0.001
Hypertension				
No	Ref		Ref	
Yes	1.37 (1.36–1.37)	< 0.001	1.05 (1.05–1.06)	< 0.001
Education	0.95 (0.95–0.95)	< 0.001	0.94 (0.94–0.94)	< 0.001
Marital status				
Married	Ref			
Divorced	2.19 (2.14–2.23)	< 0.001	-	-
Widowed	1.40 (1.39–1.41)	< 0.001	-	-
Cohabitation	-	-	-	-
Unmarried	4.04 (3.99–4.09)	< 0.001	-	-
Smoking				
Never	Ref			
Not everyday	3.76 (3.71–3.82)	< 0.001	-	-
Everyday	2.26 (2.23–2.28)	< 0.001	-	-
Smoking before but not present	4.34 (4.29–4.39)	< 0.001	-	-
Family history of osteoporosis				
No	Ref		Ref	
Unknown	1.37 (1.36–1.38)	< 0.001	1.49 (1.49–1.50)	< 0.001
Yes	1.66 (1.65–1.67)	< 0.001	1.15 (1.15–1.16)	< 0.001
Rice/pasta	0.99 (0.99–0.99)	< 0.001	-	-
Pork	-	-	0.99 (0.99–0.99)	< 0.001
Aquatic product	0.99 (0.99–0.99)	< 0.001	0.99 (0.99–0.99)	< 0.001
Dyslipidemia	1.05 (1.04–1.05)	< 0.001	1.15 (1.15–1.15)	< 0.001
β-CTX	1.02 (1.02–1.02)	< 0.001	1.02 (1.02–1.02)	< 0.001

*AOR* adjusted odds ratio, *95%CI* 95% confidence interval; “-”, the variable did not enter the multivariate logistic regression model after stepwise regression screening (*P* ≥ 0.05); β-CTX, β-crosslaps

Sensitivity analysis based on data from 1,786 participants (minimum sample size) indicated that these above factors were still related to the risk of osteoporosis and the direction of the association was consistent with the study of 2,710 participants (Supplement Table 3).

## Discussion

This study analyzed differences in the prevalence and epidemiological characteristics of osteoporosis between urban and rural populations. The prevalence of osteoporosis in urban and rural populations was 5.52% and 10.33%, respectively. Males had a lower prevalence of osteoporosis than females (2.68% vs. 13.82%). The prevalence of osteoporosis in postmenopausal women was much higher than that in premenopausal women (30.34% vs. 4.78%). In urban populations, older age, hypertension, married status (divorce, widowed, and unmarried), smoking, family history of osteoporosis, dyslipidemia, and higher β-CTX were associated with an increased risk of osteoporosis, while males, higher education level, and diet (rice/pasta and aquatic product) were related to decreased risk of osteoporosis. Similar results were also observed in rural populations.

Previous studies have reported that the prevalence of osteoporosis may differ between urban and rural populations [12, 13, 26]. The prevalence of osteoporosis in urban areas of Japan was significantly higher than that in rural areas [12]. On the contrary, our results showed that the prevalence of osteoporosis was higher in Chinese rural region than in urban region (10.33% vs. 5.22%). A meta-analysis and systematic review found that the prevalence of osteoporosis was slightly lower in urban China than in rural areas (20.87% vs. 23.92%) [14]. Maddah et al. also supported the results that women in rural areas had a significantly higher prevalence of osteoporosis than urban women [16]. Differences in the prevalence of osteoporosis in urban and rural areas between China and other countries may be related to population structure. In China, the young and strong populations tend to flock to urban areas, while older populations in rural areas may stay where they were. Our results also found that the prevalence of osteoporosis was much higher in women that in men, and in postmenopausal women than in premenopausal women. Several studies also indicated that women had a higher prevalence of osteoporosis than men [5, 27, 28]. In addition, most osteoporosis cases are reported to occur in postmenopausal women, and the incidence increases with age [15, 29]. The main reasons for the high risk of osteoporosis in women was that a significant drop in estrogen after menopause causes bone loss in women much faster than in men, and women live longer than men [30, 31].

Factors that may be related to osteoporosis in urban and rural populations were analyzed. Our results indicated that older age was linked to increased risk of osteoporosis. The relationship between age and osteoporosis may be related to the bone homeostasis, which maintained by the complex balance between bone formation and bone resorption, becomes disordered with age in adults [32]. Our results found that dyslipidemia was associated with an increased risk of osteoporosis. Dyslipidemia may cause increased oxidative stress and systemic inflammation, further leading to increased osteoclast activity and decreased bone formation [33, 34]. Higher blood pressure was found to be associated with increased risk of osteoporosis. The underlying mechanisms of the effects of high blood pressure on bone health are not fully understood [35]. This may be related to increased calcium loss due to altered calcium metabolism by elevated blood pressure [36], as well as increased sympathetic nervous system activity, enhanced inflammatory response, and altered parathyroid hormone regulation [35]. In terms of marital status, previous studies indicated that being single, divorced or widowed was associated with a higher risk of hip fracture compared with being married or cohabiting [37, 38], which supported our results. One possible explanation was that marriage provides some protection, including a complex set of environmental, social and psychological factors [39]. Consistent with our results, previous studies have also found that family history of osteoporosis was an important and independent risk factor for osteoporosis [9, 40]. In addition, males, higher education levels, higher aquatic product intake may be related to a lower risk of osteoporosis. Gender differences in osteoporosis have been reported, with women more likely to develop osteoporosis than men [41, 42]. This may be due to differences in estrogen levels between men and women, especially in postmenopausal women with significantly lower estrogen levels leading to a significantly increased risk of osteoporosis [43, 44]. Education level may also be associated with the risk of osteoporosis. Maddah et al. also found that the prevalence of osteoporosis in women with low education level was significantly higher than that in women with high education level [16]. This may be related to the fact that the more educated population was more aware of osteoporosis and had a greater awareness of disease prevention in their daily lives [45, 46]. Furthermore, Botella et al. found that increased levels of β-CTX was associated with low BMD [47], which was also consistent with our results. β-CTX is a marker of bone resorption and reflects the activity of bone cells [48]. The balance of bone formation and resorption maintains bone health, and osteoporosis occurs when bone resorption becomes more active. Both calcium and phosphorus metabolism and intake can affect BMD levels [49, 50]. Our study also showed that although serum calcium and phosphorus levels in patients with and without osteoporosis were within the normal range, there were statistical differences. However, neither calcium nor phosphorus entered the model in the multivariate logistic regression analysis. These results suggested that serum calcium and phosphorus levels were not the main factors affecting the risk of osteoporosis in the current study population.

To the best of our knowledge, the present study was the first to analyze differences in BMD and prevalence of osteoporosis between urban and rural Chinese populations. However, our study has several limitations. First, this study was a cross-sectional study, and the causal relationship between osteoporosis and influencing factors relies on prospective studies. Second, some clinical risk factors, such as medication and exposure to aromatic compounds, were not collected. These factors may influence BMD values. Third, risk factors for osteoporosis in pre- and post- menopausal women cannot be explored separately due to the low prevalence of osteoporosis in premenopausal women.

## Conclusions

The prevalence of osteoporosis in rural areas was higher than that in urban areas. In both urban and rural populations, the prevalence of osteoporosis in females was higher than that in males, and the prevalence of osteoporosis in postmenopausal females was much higher than that in premenopausal females. In addition, the factors that may be associated with the risk of osteoporosis were similar in urban and rural populations.

## Supplementary Information

**Additional file 1.**

## Data Availability

The datasets used and/or analyzed during the current study are available from the corresponding author on reasonable request.

## Abbreviations

BMD: Bone mineral density
BMI: Body mass index
SBP: Systolic blood pressure
DBP: Diastolic blood pressure
LDL-C: Low density lipoprotein cholesterol
25(OH)D: 25-Hydroxyvitamin D
PINP: Procollagen type I N-terminal propeptide
DXA: Dual energy x-ray absorptiometry
SD: Standard deviation
S.E: Standard error
