# Supplementary material for: Differences in the prevalence and risk factors of osteoporosis in chinese urban and rural regions: a cross-sectional study
Source: BMC Musculoskelet Disord. 2023 Jan 19;24:46. doi: 10.1186/s12891-023-06147-w (PMC9850530; doi:10.1186/s12891-023-06147-w)
Supplement: Supplementary file 1 — Additional file 1. [file 12891_2023_6147_MOESM1_ESM.docx]

**Supplement Table 1**. Differences in characteristics between urban participants with and without osteoporosis.

| **Variables** | **Total (n=1,363)** | **Osteoporosis** | | **Statistics** | **P** |
| --- | --- | --- | --- | --- | --- |
|  |  | **No (n=1,202)** | **Yes (n=161)** |  |  |
| Gender, n (%) |  |  |  | χ^2^=87.557 | <0.001 |
| Male | 590 (50.68) | 577 (53.16) | 13 (8.17) |  |  |
| Female | 773 (49.32) | 625 (46.84) | 148 (91.83) |  |  |
| Age (years), n (%) |  |  |  | χ^2^=87.557 | <0.001 |
| 20-29 | 91 (25.18) | 91 (26.65) | 0 (0.00) |  |  |
| 30-39 | 93 (20.29) | 92 (21.33) | 1 (2.51) |  |  |
| 40-49 | 302 (22.05) | 298 (23.10) | 4 (4.06) |  |  |
| 50-59 | 347 (15.36) | 310 (14.76) | 37 (25.76) |  |  |
| 60-69 | 407 (9.75) | 318 (8.34) | 89 (33.81) |  |  |
| 70-79 | 113 (6.43) | 84 (5.01) | 29 (30.74) |  |  |
| 80-89 | 10 (0.94) | 9 (0.81) | 1 (3.12) |  |  |
| BMI (kg/m^2^), mean (S.E) | 24.12 (0.27) | 24.08 (0.28) | 24.84 (0.50) | t=-1.33 | 0.183 |
| BMI (kg/m^2^), n (%) |  |  |  | χ^2^=0.591 | 0.744 |
| Underweight (<18.5) | 22 (2.37) | 18 (2.35) | 4 (2.75) |  |  |
| Normal (18.5-23.9) | 532 (52.02) | 455 (52.28) | 77 (47.56) |  |  |
| Overweight (≥24.0) | 809 (45.61) | 729 (45.37) | 80 (49.70) |  |  |
| SBP (mmHg), mean (S.E) | 124.00 (1.36) | 123.21 (1.39) | 137.54 (2.67) | t=-4.76 | <0.001 |
| DBP (mmHg), mean (S.E) | 75.98 (0.95) | 75.98 (1.01) | 75.95 (1.21) | t=0.02 | 0.985 |
| Hypertension, n (%) |  |  |  | χ^2^=29.540 | <0.001 |
| No | 891 (77.16) | 805 (78.60) | 86 (52.67) |  |  |
| Yes | 472 (22.84) | 397 (21.40) | 75 (47.33) |  |  |
| Heart rate (BPM), mean (S.E) | 73.50 (0.54) | 73.55 (0.57) | 72.77 (1.24) | t=0.56 | 0.573 |
| Nation, n (%) |  |  |  | χ^2^=0.001 | 0.977 |
| Han | 1358 (99.80) | 1198 (99.80) | 160 (99.80) |  |  |
| Others | 5 (0.20) | 4 (0.20) | 1 (0.20) |  |  |
| Education level, n (%) |  |  |  | χ^2^=71.731 | <0.001 |
| <high school | 865 (36.07) | 725 (33.39) | 140 (82.01) |  |  |
| High school | 255 (25.89) | 240 (26.76) | 15 (10.91) |  |  |
| College and above | 243 (38.04) | 237 (39.85) | 6 (7.08) |  |  |
| Marital status, n (%) |  |  |  | χ^2^=135.588 | <0.001 |
| Unmarried | 51 (14.76) | 50 (15.47) | 1 (2.51) |  |  |
| Married | 1237 (82.08) | 1099 (82.28) | 138 (78.59) |  |  |
| Cohabitation | 3 (0.33) | 3 (0.35) | 0 (0.00) |  |  |
| Widowed | 60 (2.21) | 39 (1.29) | 21 (18.04) |  |  |
| Divorced | 12 (0.63) | 11 (0.62) | 1 (0.87) |  |  |
| Income ($), mean (S.E) | 13129.94 (384.79) | 13388.20 (409.11) | 8709.71 (609.68) | t=6.37 | <0.001 |
| Expenditure ($), mean (S.E) | 7374.94 (266.61) | 7536.18 (279.12) | 4615.26 (338.68) | t=6.66 | <0.001 |
| Smoking, n (%) |  |  |  | χ^2^=8.290 | 0.040 |
| Everyday | 228 (20.54) | 221 (21.34) | 7 (6.84) |  |  |
| Not everyday | 39 (3.34) | 36 (3.35) | 3 (3.21) |  |  |
| Smoking before but not present | 104 (5.17) | 100 (5.26) | 4 (3.68) |  |  |
| Never | 992 (70.95) | 845 (70.06) | 147 (86.27) |  |  |
| Drinking, n (%) |  |  |  | χ^2^=8.290 | 0.040 |
| Never | 863 (60.79) | 732 (59.52) | 131 (82.49) |  |  |
| Sometimes | 320 (30.02) | 297 (30.91) | 23 (14.82) |  |  |
| Often but not exceeding the norm | 127 (6.54) | 120 (6.76) | 7 (2.69) |  |  |
| Often and beyond the norm | 53 (2.66) | 53 (2.81) | 0 (0.00) |  |  |
| BMD |  |  |  |  |  |
| Lumbar spine L1 (g/cm2), mean (S.E) | 0.95 (0.01) | 0.96 (0.01) | 0.69 (0.02) | t=15.75 | <0.001 |
| Lumbar spine L2 (g/cm2), mean (S.E) | 1.02 (0.01) | 1.04 (0.01) | 0.71 (0.01) | t=20.03 | <0.001 |
| Lumbar spine L3 (g/cm2), mean (S.E) | 1.09 (0.01) | 1.11 (0.01) | 0.76 (0.02) | t=16.87 | <0.001 |
| Lumbar spine L4 (g/cm2), mean (S.E) | 1.08 (0.01) | 1.09 (0.01) | 0.79 (0.02) | t=12.55 | <0.001 |
| Greater trochanter (g/cm2), mean (S.E) | 0.65 (0.01) | 0.66 (0.01) | 0.47 (0.01) | t=14.40 | <0.001 |
| Total hip (g/cm2), mean (S.E) | 0.88 (0.01) | 0.90 (0.01) | 0.66 (0.01) | t=15.73 | <0.001 |
| Family history of osteoporosis, n (%) |  |  |  | χ^2^=14.462 | <0.001 |
| Yes | 95 (6.89) | 84 (6.82) | 11 (8.01) |  |  |
| No | 1005 (78.26) | 895 (79.20) | 110 (62.29) |  |  |
| Unknown | 263 (14.85) | 223 (13.98) | 40 (29.70) |  |  |
| Diet |  |  |  |  |  |
| Rice/pasta (g/day), mean (S.E) | 524.41 (78.82) | 534.47 (83.05) | 352.51 (23.80) | t=2.11 | 0.035 |
| Tuber (g/day), mean (S.E) | 327.58 (13.38) | 329.21 (14.06) | 298.68 (24.32) | t=1.09 | 0.277 |
| Pork (g/day), mean (S.E) | 261.95 (8.83) | 263.39 (9.24) | 236.48 (22.81) | t=1.09 | 0.274 |
| Aquatic product (g/day), mean (S.E) | 408.61 (62.27) | 417.26 (65.54) | 260.50 (17.48) | t=2.31 | 0.021 |
| Vegetables (g/day), mean (S.E) | 507.04 (18.12) | 505.73 (19.06) | 529.33 (31.65) | t=-0.64 | 0.523 |
| Eggs (g/day), mean (S.E) | 174.23 (5.87) | 174.46 (6.19) | 170.33 (8.65) | t=0.39 | 0.698 |
| Physical activity |  |  |  |  |  |
| High-intensity, n (%) | 203 (9.24) | 179 (9.11) | 24 (11.41) | χ^2^=0.655 | 0.418 |
| Moderate-intensity, n (%) | 859 (56.54) | 752 (55.89) | 107 (67.72) | χ^2^=3.119 | 0.077 |
| Activity duration (min), mean (S.E) | 218.67 (20.91) | 221.40 (22.05) | 171.88 (16.40) | t=1.80 | 0.072 |
| Sleep duration, mean (S.E) | 450.17 (5.32) | 451.14 (5.60) | 433.55 (9.93) | t=1.54 | 0.123 |
| Fasting plasma glucose (mmol/L), mean (S.E) | 5.45 (0.06) | 5.43 (0.06) | 5.84 (0.17) | t=-2.28 | 0.023 |
| Triglyceride (mmol/L), mean (S.E) | 1.61 (0.07) | 1.61 (0.08) | 1.57 (0.07) | t=0.35 | 0.726 |
| Total cholesterol (mmol/L), mean (S.E) | 4.44 (0.08) | 4.40 (0.08) | 5.13 (0.11) | t=-5.20 | <0.001 |
| LDL-C (mmol/L), mean (S.E) | 2.63 (0.06) | 2.59 (0.06) | 3.22 (0.10) | t=-5.29 | <0.001 |
| HDL-C (mmol/L), mean (S.E) | 1.29 (0.02) | 1.28 (0.02) | 1.50 (0.05) | t=-4.20 | <0.001 |
| Hyperglycemia, n (%) |  |  |  | χ^2^=6.832 | 0.009 |
| No | 1059 (87.33) | 932 (87.88) | 127 (77.92) |  |  |
| Yes | 304 (12.67) | 270 (12.12) | 34 (22.08) |  |  |
| Dyslipidemia, n (%) |  |  |  | χ^2^=21.893 | <0.001 |
| No | 497 (52.32) | 450 (53.79) | 47 (27.12) |  |  |
| Yes | 866 (47.68) | 752 (46.21) | 114 (72.88) |  |  |
| Calcium (mmol/L), mean (S.E) | 2.33 (0.01) | 2.33 (0.01) | 2.39 (0.02) | t=-2.70 | 0.007 |
| Phosphorus (mmol/L), mean (S.E) | 1.16 (0.01) | 1.16 (0.01) | 1.21 (0.02) | t=-2.80 | 0.005 |
| 25(OH)D (ng/ml), mean (S.E) | 20.92 (0.52) | 20.96 (0.55) | 20.22 (0.66) | t=0.87 | 0.387 |
| β-CTX (ng/ml), mean (S.E) | 68.50 (1.58) | 67.59 (1.66) | 84.04 (1.82) | t=-6.68 | <0.001 |
| PINP (ng/ml), mean (S.E) | 0.30 (0.01) | 0.29 (0.01) | 0.40 (0.02) | t=-4.80 | <0.001 |

Note: BMI, body mass index; SBP, systolic blood pressure; DBP, diastolic blood pressure; BPM, beet per minute; BMD, bone mineral density; LDL-C, low density lipoprotein cholesterol; HDL-C, high density lipoprotein cholesterol; 25(OH)D, 25-hydroxyvitamin D; β-CTX, β-crosslaps; PINP, procollagen type I N-terminal propeptide.

**Supplement Table 2**. Differences in characteristics between rural participants with and without osteoporosis.

| **Variables** | **Total (n=1,348)** | **Osteoporosis** | | **Statistics** | **P** |
| --- | --- | --- | --- | --- | --- |
|  |  | **No (n=1,138)** | **Yes (n=210)** |  |  |
| Gender, n (%) |  |  |  | χ^2^=17.080 | <0.001 |
| Male | 581 (48.67) | 553 (52.08) | 28 (19.05) |  |  |
| Female | 767 (51.33) | 585 (47.92) | 182 (80.95) |  |  |
| Age (years), n (%) |  |  |  | χ^2^=86.194 | <0.001 |
| 20-29 | 90 (18.50) | 87 (20.10) | 3 (4.60) |  |  |
| 30-39 | 91 (18.05) | 88 (19.07) | 3 (9.19) |  |  |
| 40-49 | 276 (24.18) | 266 (26.12) | 10 (7.38) |  |  |
| 50-59 | 339 (17.50) | 296 (17.62) | 43 (16.50) |  |  |
| 60-69 | 391 (12.02) | 296 (10.24) | 95 (27.42) |  |  |
| 70-79 | 142 (8.71) | 93 (6.04) | 49 (31.88) |  |  |
| 80-89 | 19 (1.04) | 12 (0.81) | 7 (3.04) |  |  |
| BMI (kg/m^2^), mean (S.E) | 24.54 (0.16) | 24.70 (0.17) | 23.13 (0.30) | t=4.53 | <0.001 |
| BMI (kg/m^2^), n (%) |  |  |  | χ^2^=10.127 | 0.006 |
| Underweight (<18.5) | 36 (3.82) | 22 (3.21) | 14 (9.11) |  |  |
| Normal (18.5-23.9) | 546 (41.72) | 443 (40.75) | 103 (50.11) |  |  |
| Overweight (≥24.0) | 766 (54.47) | 673 (56.04) | 93 (40.79) |  |  |
| SBP (mmHg), mean (S.E) | 131.63 (0.79) | 130.68 (0.84) | 139.89 (1.86) | t=-4.51 | <0.001 |
| DBP (mmHg), mean (S.E) | 78.76 (0.46) | 78.63 (0.49) | 79.87 (1.43) | t=-0.82 | 0.412 |
| Hypertension, n (%) |  |  |  | χ^2^=19.568 | <0.001 |
| No | 791 (68.32) | 689 (70.58) | 102 (48.75) |  |  |
| Yes | 557 (31.68) | 449 (29.42) | 108 (51.25) |  |  |
| Heart rate (BPM), mean (S.E) | 73.95 (0.41) | 74.04 (0.44) | 73.18 (0.93) | t=0.83 | 0.409 |
| Education level, n (%) |  |  |  | χ^2^=33.909 | <0.001 |
| <high school | 1036 (60.97) | 842 (57.84) | 194 (88.06) |  |  |
| High school | 171 (16.72) | 161 (18.08) | 10 (4.92) |  |  |
| College and above | 141 (22.32) | 135 (24.08) | 6 (7.02) |  |  |
| Marital status, n (%) |  |  |  | χ^2^=73.252 | <0.001 |
| Unmarried | 42 (7.48) | 41 (8.22) | 1 (0.99) |  |  |
| Married | 1153 (86.15) | 986 (86.73) | 167 (81.16) |  |  |
| Cohabitation | 58 (2.47) | 48 (2.37) | 10 (3.33) |  |  |
| Widowed | 90 (3.42) | 58 (2.14) | 32 (14.53) |  |  |
| Divorced | 5 (0.48) | 5 (0.54) | 0 (0.00) |  |  |
| Income ($), mean (S.E) | 14406.19 (436.58) | 14936.06 (474.09) | 9807.97 (824.71) | t=5.39 | <0.001 |
| Expenditure ($), mean (S.E) | 8110.75 (227.07) | 8395.56 (246.39) | 5639.20 (394.17) | t=5.93 | <0.001 |
| Smoking, n (%) |  |  |  | χ^2^=36.782 | <0.001 |
| Everyday | 289 (23.78) | 273 (25.73) | 16 (6.86) |  |  |
| Not everyday | 30 (2.19) | 28 (2.34) | 2 (0.93) |  |  |
| Smoking before but not present | 77 (4.38) | 71 (4.59) | 6 (2.58) |  |  |
| Never | 952 (69.64) | 766 (67.34) | 186 (89.63) |  |  |
| Drinking, n (%) |  |  |  | χ^2^=30.808 | <0.001 |
| Never | 957 (71.13) | 778 (69.17) | 179 (88.17) |  |  |
| Sometimes | 198 (16.66) | 183 (17.88) | 15 (6.08) |  |  |
| Often but not exceeding the norm | 144 (8.74) | 130 (9.16) | 14 (5.09) |  |  |
| Often and beyond the norm | 49 (3.47) | 47 (3.79) | 2 (0.66) |  |  |
| BMD |  |  |  |  |  |
| Lumbar spine L1 (g/cm2), mean (S.E) | 0.87 (0.01) | 0.90 (0.01) | 0.62 (0.01) | t=21.10 | <0.001 |
| Lumbar spine L2 (g/cm2), mean (S.E) | 0.96 (0.01) | 0.99 (0.01) | 0.68 (0.01) | t=24.51 | <0.001 |
| Lumbar spine L3 (g/cm2), mean (S.E) | 1.02 (0.01) | 1.05 (0.01) | 0.73 (0.01) | t=24.64 | <0.001 |
| Lumbar spine L4 (g/cm2), mean (S.E) | 1.03 (0.01) | 1.06 (0.01) | 0.75 (0.01) | t=22.70 | <0.001 |
| Greater trochanter (g/cm2), mean (S.E) | 0.63 (0.00) | 0.65 (0.00) | 0.44 (0.01) | t=20.22 | <0.001 |
| Total hip (g/cm2), mean (S.E) | 0.87 (0.01) | 0.90 (0.01) | 0.64 (0.01) | t=21.46 | <0.001 |
| Family history of osteoporosis, n (%) |  |  |  | χ^2^=17.341 | <0.001 |
| Yes | 68 (4.61) | 56 (4.51) | 12 (5.45) |  |  |
| No | 946 (76.91) | 821 (78.73) | 125 (61.05) |  |  |
| Unknown | 334 (18.49) | 261 (16.76) | 73 (33.50) |  |  |
| Diet |  |  |  |  |  |
| Rice/pasta (g/day), mean (S.E) | 294.69 (7.03) | 297.06 (7.27) | 273.98 (25.82) | t=0.86 | 0.390 |
| Tuber (g/day), mean (S.E) | 300.83 (9.71) | 299.90 (10.58) | 308.79 (20.42) | t=-0.39 | 0.699 |
| Pork (g/day), mean (S.E) | 232.43 (6.60) | 236.77 (7.22) | 194.15 (11.02) | t=3.23 | 0.001 |
| Aquatic product (g/day), mean (S.E) | 337.80 (19.66) | 348.97 (21.72) | 239.56 (13.85) | t=4.25 | <0.001 |
| Vegetables (g/day), mean (S.E) | 518.62 (11.71) | 516.74 (12.31) | 534.91 (37.22) | t=-0.46 | 0.643 |
| Eggs (g/day), mean (S.E) | 185.02 (3.65) | 183.97 (3.42) | 194.18 (18.91) | t=-0.53 | 0.595 |
| Physical activity |  |  |  |  |  |
| High-intensity, n (%) | 189 (15.48) | 176 (15.79) | 13 (12.75) | χ^2^=0.200 | 0.654 |
| Moderate-intensity, n (%) | 1053 (73.34) | 873 (72.44) | 180 (81.17) | χ^2^=1.526 | 0.217 |
| Activity duration (min), mean (S.E) | 255.38 (8.07) | 253.99 (8.41) | 267.45 (27.02) | t=-0.48 | 0.634 |
| Fasting plasma glucose (mmol/L), mean (S.E) | 5.12 (0.07) | 5.10 (0.07) | 5.36 (0.14) | t=-1.63 | 0.103 |
| Triglyceride (mmol/L), mean (S.E) | 1.65 (0.09) | 1.65 (0.10) | 1.63 (0.08) | t=0.15 | 0.880 |
| Total cholesterol (mmol/L), mean (S.E) | 4.79 (0.04) | 4.75 (0.04) | 5.13 (0.08) | t=-4.13 | <0.001 |
| LDL-C (mmol/L), mean (S.E) | 2.83 (0.03) | 2.80 (0.04) | 3.08 (0.09) | t=-2.92 | 0.004 |
| HDL-C (mmol/L), mean (S.E) | 1.41 (0.01) | 1.39 (0.02) | 1.57 (0.03) | t=-5.25 | <0.001 |
| Hyperglycemia, n (%) |  |  |  | χ^2^=0.118 | 0.731 |
| No | 1023 (78.07) | 868 (78.21) | 155 (76.79) |  |  |
| Yes | 325 (21.93) | 270 (21.79) | 55 (23.21) |  |  |
| Dyslipidemia, n (%) |  |  |  | χ^2^=8.931 | 0.003 |
| No | 522 (46.59) | 458 (48.21) | 64 (32.54) |  |  |
| Yes | 826 (53.41) | 680 (51.79) | 146 (67.46) |  |  |
| Calcium (mmol/L), mean (S.E) | 2.40 (0.00) | 2.39 (0.00) | 2.42 (0.01) | t=-1.98 | 0.048 |
| Phosphorus (mmol/L), mean (S.E) | 1.17 (0.01) | 1.16 (0.01) | 1.24 (0.01) | t=-4.63 | <0.001 |
| 25(OH)D (ng/ml), mean (S.E) | 23.02 (0.29) | 23.00 (0.32) | 23.14 (0.61) | t=-0.19 | 0.849 |
| β-CTX (ng/ml), mean (S.E) | 74.60 (0.82) | 73.13 (0.87) | 87.29 (2.03) | t=-6.42 | <0.001 |
| PINP (ng/ml), mean (S.E) | 0.33 (0.01) | 0.33 (0.01) | 0.42 (0.01) | t=-5.41 | <0.001 |

Note: BMI, body mass index; SBP, systolic blood pressure; DBP, diastolic blood pressure; BPM, beet per minute; BMD, bone mineral density; LDL-C, low density lipoprotein cholesterol; HDL-C, high density lipoprotein cholesterol; 25(OH)D, 25-hydroxyvitamin D; β-CTX, β-crosslaps; PINP, procollagen type I N-terminal propeptide.

**Supplement Table 3**. The sensitivity analysis was based on data from 1,786 participants.

| **Variables** | **Urban** | | **Rural** | |
| --- | --- | --- | --- | --- |
|  | **AOR (95%CI)** | ***P*** | **AOR (95%CI)** | ***P*** |
| Gender |  |  |  |  |
| Male | 0.03 (0.03-0.03) | <0.001 | 0.29 (0.29-0.30) | <0.001 |
| Female | Ref |  | Ref |  |
| Age | 2.53 (2.52-2.54) | <0.001 | 1.44 (1.44-1.44) | <0.001 |
| Hypertension |  |  |  |  |
| No | Ref |  | Ref |  |
| Yes | 1.26 (1.25-1.27) | <0.001 | 1.19 (1.19-1.20) | <0.001 |
| Education | 0.97 (0.97-0.98) | <0.001 | 0.80 (0.80-0.80) | <0.001 |
| Marital status |  |  |  |  |
| Married | Ref |  |  |  |
| Divorced | - | - | - | - |
| Widowed | 1.49 (1.48-1.51) | <0.001 | - | - |
| Cohabitation | - | - | - | - |
| Unmarried | 7.73 (7.62-7.84) | <0.001 | - | - |
| Smoking |  |  |  |  |
| Never | Ref |  |  |  |
| Not everyday | 5.39 (5.31-5.48) | <0.001 | - | - |
| Everyday | 5.80 (5.74-5.85) | <0.001 | - | - |
| Smoking before but not present | 4.79 (4.72-4.86) | <0.001 | - | - |
| Family history of osteoporosis |  |  |  |  |
| No | Ref |  | Ref |  |
| Unknown | 1.31 (1.31-1.32) | <0.001 | 1.88 (1.88-1.89) | <0.001 |
| Yes | 1.60 (1.58-1.61) | <0.001 | 1.50 (1.49-1.50) | <0.001 |
| Rice/pasta | 0.99 (0.99-0.99) | <0.001 | - | - |
| Pork | - | - | 0.99 (0.99-0.99) | <0.001 |
| Aquatic product | 0.99 (0.99-0.99) | <0.001 | 0.99 (0.99-0.99) | <0.001 |
| Dyslipidemia | 1.10 (1.09-1.10) | <0.001 | 1.24 (1.24-1.25) | <0.001 |
| β-CTX | 1.02 (1.02-1.02) | <0.001 | 1.02 (1.02-1.02) | <0.001 |

Note: AOR, adjusted odds ratio; 95%CI, 95% confidence interval; “-”, the variable did not enter the multivariate logistic regression model after stepwise regression screening; β-CTX, β-crosslaps.
